# Supplementary material for: Enhancing national cholera surveillance using rapid diagnostic tests (RDTs): A mixed methods evaluation
Source: PLoS Negl Trop Dis. 2025 May 6;19(5):e0013019. doi: 10.1371/journal.pntd.0013019 (PMC12077796; doi:10.1371/journal.pntd.0013019)
Supplement: S1 Table — (DOCX) [file pntd.0013019.s001.docx]

**Supplemental Materials**

**S1 Table. Open-coding Framework sub-theme definitions for Government interviews.**

| A. Job description | Unlike with the lab and physician transcripts, government stakeholders will have varied roles. While this information is not a "theme" and will not be analyzed thematically, it will be used for understanding of the background/context from which the interview subjects are speaking. Record brief but relevant description of subject's job/role especially as it relates to cholera surveillance. |
| --- | --- |
| B. Barriers | Record any details on subjects’ perspective on critical barriers to the implementation of RDT surveillance at sites. Examples may include perspectives on addition of cholera RDTs to cholera testing and surveillance protocols/algorithms, critical barriers to RDT implementation at sites, degree to which facility-level activities like ordering tests or reporting results are siloed vs. integrated, disincentives to perform/interpret/order tests, availability of tests or physicians or lab personnel etc. |
| C. Facilitators | Record any details on subjects’ perspective on critical success factors to the implementation of RDT surveillance at sites. Examples may include perspectives on addition of cholera RDTs to cholera testing and surveillance protocols/algorithms, critical facilitators to RDT implementation at sites, degree to which facility-level activities like ordering tests or reporting results are siloed vs. integrated, incentives to perform/interpret/order tests, availability of tests or physicians or lab personnel. |
| D. Fidelity and Fit | Record any details on subjects’ perspective on: 1) If integration and ordering of cholera RDTs at surveillance sites were implemented as intended; 2) How and why changes were made to the implementation strategy in response to the context. Examples could include how/if any material (i.e., resources, time, workload, etc.), cultural (i.e., work environment, interpersonal/ partnership/stakeholder/implementer dynamics etc.) or contextual factors (i.e., epidemiological changes, politics etc.) external to RISE impacted RDT implementation; 3) If/How RDT implementation strategies , distribution, training and data integration (i.e., printed aids, PPHL meeting) were appropriate/effective for Nepali cholera surveillance and detection. |
| E. Nepali Cholera Surveillance System - Structure and Reporting | Record any information shared by subject on the structure of the Nepali infectious disease surveillance system, or how information is reported within that system. We are primarily interested in the system as it relates to cholera, but also include relevant comparisons/references made to other disease surveillance/reporting mechanisms. Examples could include surveillance or reporting guidelines or processes (e.g., steps of outbreak declaration), major infrastructure, procedures or hierarchies of different entities involved in surveillance activities, modes of reporting (e.g., electronic, by phone), differences by geographic region or facility type etc. |
| F. Lab Diagnostics for Pathogen Detection/Confirmation OTHER than the Cholera RDT | Record any information shared by subject on procedures or methods of testing pathogens for confirmation in the context of surveillance - other than cholera RDTs. We are primarily interested in detection/confirmation as it relates to cholera, but also include relevant comparisons/references made to other pathogens. Examples may include types of tests performed (e.g., Cary Blair, PCR, serology) and why certain methods are preferred, challenges/advantages of different methods, logistics associated with confirmation processes etc. |
| G. Roles in cholera surveillance | Record any details on subjects’ perspectives on their roles or the roles of others in cholera surveillance in Nepal. |
| H. POV on Negatives of RDT Use | Include any information on subjects' thoughts on the challenges/limitations/drawbacks of using cholera RDTs in Nepal. Examples could include implications for screening, surveillance and disease control, workload, reporting, logistics, relative barriers for different facility types or geographical areas, seasonality, private vs. public sector factors, cost to patients/government, specificity and sensitivity, patient care etc. |
| I. POV on Benefits of RDT Use | Include any information on subjects' thoughts on the benefits of using cholera RDTs in Nepal. Examples could include implications for screening, surveillance and disease control, workload, reporting, logistics, relative facilitators for different facility types or geographical areas, seasonality, private vs. public sector factors, cost to patients/government, specificity and sensitivity, patient care etc. |
| J. Additional Recommendations | Record information on additional recommendations subjects have about cholera RDT use in Nepal. Examples could include suggestions for monitoring and evaluation, edits to cholera outbreak protocols/algorithms, training, rollout and scale-up, government oversight, distribution, job aids, sensitization etc. |
| K. Other content, Comments, Questions, Follow-ups | Record information that doesn't fit into the other columns, but seems important/relevant to the overall study, questions you may have that need to be followed-up on, suggestions from subjects that should be followed-up on etc. |
