# Supplementary material for: Enhancing national cholera surveillance using rapid diagnostic tests (RDTs): A mixed methods evaluation
Source: PLoS Negl Trop Dis. 2025 May 6;19(5):e0013019. doi: 10.1371/journal.pntd.0013019 (PMC12077796; doi:10.1371/journal.pntd.0013019)
Supplement: S2 Table — (DOCX) [file pntd.0013019.s002.docx]

**S2 Table. Open-coding Framework sub-theme definitions for Physician interviews.**

| A. Barriers | Record any details on 1) Critical barriers to RDT implementation at surveillance sites. Examples could include degree to which facility-level activities like ordering tests or reporting results are siloed vs. integrated, disincentives to perform/interpret tests or disinterest, availability of tests or lab personnel. |
| --- | --- |
| B. Facilitators | Record any details on 1) Critical success factors to RDT implementation at surveillance sites. Examples could include degree to which facility-level activities like ordering tests or receiving results are integrated vs. siloed, availability of tests or lab personnel. |
| C. Fidelity and Fit | Record any details on 1) If integration and ordering of cholera RDTs at surveillance sites were implemented as intended; 2) How and why changes were made to the implementation strategy in response to the context. Examples could include how/if any material (i.e., resources, time, workload, etc.), cultural (i.e., work environment, interpersonal/ partnership/stakeholder/implementer dynamics etc.) or contextual factors (i.e., epidemiological changes, politics etc.) external to RISE impacted RDT implementation; 3) If/How RDT implementation strategies , distribution, training and data integration (i.e., printed aids, PPHL meeting) were appropriate/effective for Nepali cholera surveillance and detection. |
| D. Experiences performing cholera RDTs | Record any details on physicians’ experiences with the process of ordering cholera RDTs or the process of receiving test results. Examples could include their thoughts on ordering protocols, process of ordering a cholera RDT including as it relates to patient interactions (i.e., any forms to complete, patient counseling, getting a sample etc.). |
| E. Experiences diagnosing cholera | Record any details on physicians’ experiences diagnosing cholera. Examples could include their thoughts on symptoms, decision-making process as it relates to of diagnosis vs. treatment and/or surveillance, utility of or reasons/motivations to diagnose, options for diagnosis as it relates to different types of tests and reasons for selecting one over another, decision-making process as it relates to deciding if it’s a suspect cholera case and the decision to test or treat based on that determination. |
| F. Roles in cholera surveillance | Record any details on physicians’ perspectives on their roles or the roles of others in cholera surveillance in Nepal. |
| G. POV on Negatives of RDT Use | Include any information physicians' thoughts on the challenges/limitations/drawbacks of using cholera RDTs in Nepal. Examples could include implications for screening, surveillance and disease control, workload, reporting, logistics, relative barriers for different facility types or geographical areas, seasonality, private vs. public sector factors, cost to patients/government, specificity and sensitivity, patient care etc. |
| H. POV on Benefits of RDT Use | Include any information physicians' thoughts on the benefits of using cholera RDTs in Nepal. Examples could include implications for screening, surveillance and disease control, workload, reporting, logistics, relative benefits to different facility types or geographical areas, seasonality, cost to patients/government, specificity and sensitivity, patient care etc. |
| I. Additional Recommendations | Record information on additional recommendations physicians have about cholera RDT use in Nepal. Examples could include suggestions for training, rollout and scale-up, government oversight, distribution, job aids, sensitization etc. |
| J. Other content, Comments, Questions, Follow-ups | Record information that doesn't fit into the other columns, but seems important/relevant to the overall study, questions you may have that need to be followed-up on, suggestions from subjects that should be followed-up on etc. |
