# Supplementary material for: Enhancing national cholera surveillance using rapid diagnostic tests (RDTs): A mixed methods evaluation
Source: PLoS Negl Trop Dis. 2025 May 6;19(5):e0013019. doi: 10.1371/journal.pntd.0013019 (PMC12077796; doi:10.1371/journal.pntd.0013019)
Supplement: S3 Table — (DOCX) [file pntd.0013019.s003.docx]

**S3 Table. Open-coding Framework sub-theme definitions for Laboratory Technician interviews.**

| A. Barriers | Record any details on 1) Critical barriers to RDT implementation at surveillance sites. Examples could include degree to which facility-level activities like reporting results are siloed vs. integrated, disincentives to perform/interpret tests or disinterest, availability of tests. |
| --- | --- |
| B. Facilitators | Record any details on 1) Critical success factors to RDT implementation at surveillance sites. Examples could include degree to which facility-level activities like reporting results are integrated vs. siloed, availability of tests. |
| C. Fidelity and Fit | Record any details on 1) If distribution and integration of cholera RDTs at surveillance sites were implemented as intended; 2) How and why changes were made to the implementation strategy in response to the context. Examples could include how/if any material (i.e., resources, time, workload, etc.), cultural (i.e., work environment, interpersonal/ partnership/stakeholder/implementer dynamics etc.) or contextual factors (i.e., epidemiological changes, politics etc.) external to RISE impacted RDT implementation; 3) If/How RDT implementation strategies , distribution, training and data integration (i.e., printed aids, PPHL meeting) were appropriate/effective for Nepali cholera surveillance and detection. |
| D. Experiences performing cholera RDTs | Record any details on laboratorians’ experiences performing cholera RDTs. Examples could include their thoughts on testing protocols, ease of use (may be relative to other methods of testing), what would make the tests easier and more convenient to perform etc. |
| E. Experiences interpreting cholera RDTs | Record any details on laboratorians’ experiences interpreting cholera RDTs. Examples could include their thoughts on reading test results (may be relative to other methods of testing), false positives, diagnosis, application to outbreak algorithms, what would make the tests easier and more convenient to interpret etc. |
| F. Roles in cholera surveillance | Record any details on laboratorians’ perspectives on their roles or the roles of others in cholera surveillance in Nepal. |
| G. POV on Negatives of RDT Use | Include any information laboratorians thoughts on the challenges/limitations/drawbacks of using cholera RDTs in Nepal. Examples could include implications for screening, surveillance and disease control, workload, reporting, logistics, relative barriers for different facility types or geographical areas, seasonality, private vs. public sector factors, cost to patients/government, specificity and sensitivity etc. |
| H. POV on Benefits of RDT Use | Include any information laboratorians thoughts on the benefits of using cholera RDTs in Nepal. Examples could include implications for screening, surveillance and disease control, workload, reporting, logistics, relative benefits to different facility types or geographical areas, seasonality, cost to patients/government, specificity and sensitivity etc. |
| I. Additional Recommendations | Record information on additional recommendations laboratorians have about cholera RDT use in Nepal. Examples could include suggestions for training, rollout and scale-up, government oversight, distribution, job aids, sensitization etc. |
| J. Other content, Comments, Questions, Follow-ups | Record information that doesn't fit into the other columns, but seems important/relevant to the overall study, questions you may have that need to be followed-up on, suggestions from subjects that should be followed-up on etc. |
